# Prevalence and Factors Associated With Suicidal Ideation Among Adult Eritrean Refugees in Northern Ethiopia

**DOI:** 10.3389/fpubh.2022.841848

**Published:** 2022-05-04

**Authors:** Teferi Gebru Gebremeskel, Mulaw Berhe, Tadis Brhane Tesfahunegn, Hailay Abrha Gesesew, Paul R. Ward

**Affiliations:** ^1^Department of Reproductive Health, College of Health Sciences, Aksum University, Aksum, Ethiopia; ^2^Discipline of Public Health, Flinders University, Adelaide, SA, Australia; ^3^Department of Public Health, College of Health Sciences, Aksum University, Aksum, Ethiopia; ^4^Centre for Research on Health Policy, Torrens University, Adelaide, SA, Australia; ^5^Departments of Epidemiology, College of Health Sciences, Mekelle University, Mekelle, Ethiopia

**Keywords:** suicidal ideation, refugee, Eritrea, Tigray, Ethiopia

## Abstract

**Background:**

The present study assessed the prevalence of and factor associated with suicidal ideations among adult Eritrean refugees in Tigray, Ethiopia.

**Methods:**

A community-based cross-sectional study was carried out among 400 adult refugees living in the Mai-Aini refugee camp in Tigray, Northern Ethiopia from September 2019 to May 2020. The response variable was suicidal ideation and was measured using World Mental Health (WMH) Survey Initiative Version of the World Health Organization Composite International Diagnostic Interview. We applied bivariate and multivariate logistic regression to determine predictors for suicide ideations. Odds ratios and *p*-values were determined to check the associations between variables, and a *p*-value <0.05 was considered as a cut-off for statistical significance.

**Results:**

The prevalence of suicidal ideations was 20.5% (95% CI: 16.4%, 24.5%). Having previous history of trauma **[**AOR = 2.3, 95% CI: 1.4, 4.5**]**, a history of chronic illness **[**AOR = 2.9, 95% CI: 1.3, 6.5**]**, a family history of mental disorder **[**AOR = 3.08, 95% CI: 1.3, 7.06**]**, and history of post-traumatic stress disorder **[**AOR = 5.7, 95% CI: 2.8, 11.5**]** were significantly associated with suicidal ideations.

**Conclusions:**

This study showed that during the stay in the refugee camp, there was a high prevalence of suicide ideations compared to the prevalence of suicide ideations among the general populations of Ethiopia, Europe, and China, and the lifetime pooled prevalence across 17 countries. Having previous history of trauma, a history of chronic illness, a family history of mental disorder, and history of post-traumatic stress disorder were the factors statistically associated with the suicidal ideation.

## Introduction

Suicidal ideation is defined as thoughts, ideas, and the desire to commit suicide, being a frequent behavior among individuals and characterized as a personality disorder or with the character of emotion (1). Most suicidal people do not want to die; they just do not want to live with the pain they are feeling (2). Helping a suicidal person talk about their thoughts and feelings can help save a life (2).

According to the World Health Organization (WHO), a person commits suicide every 40 seconds anywhere in the world, and a person tries to die every 3 seconds. When a person commits suicide, there is a serious psychological, social, and financial impact on at least six other people (3). The World Health Organization estimates that 800,000 people commit suicide each year, with 11.4 deaths per 100,000 globally (4). Suicide, accounting 1% of the global disease burden (3), is a major public health problem and the rates have risen by 60% over the last 45 years.

Evidence shows that 85% of suicidal deaths occurred in low and middle-income countries (5), and WHO estimates that each suicidal rate in developing countries is relatively high compared to developed countries (6). In particular, the 12 month suicidal ideation estimates, plans, and trials are 2.0, 0.6, and 0.3% for developed countries, and 2.1, 0.7, and 0.4% for developing countries, respectively (6). Suicide rates in China are estimated at 20% of worldwide (7). Suicidal thoughts, suicide, and suicide among children and young people are a major global concern, especially in refugee settings (5).

The risk of each suicidal behavior is significantly related to being female, residence, younger age, having fewer years of formal education, before ever being married, unemployment, mental disorders (especially depression); moments of crisis; chronic pain and illness; weak family and social relationships, conflict, disaster, violence, abuse, and loss; substantial geographic variations; being a refugee, prior suicide ideation (6, 8–12).

Literature shows that refugees are at high risk of distress and subsequently at an elevated rate of suicidal death (13). For example, Leiler et al. presented that nearly 30% of participants were bothered by suicidal ideation at some frequency, and 18% of participants had thoughts of suicidal ideation several days (13). In the WHO Mental Health Action Plan 2013–2030, WHO Member States have committed themselves to working toward the global target of reducing the suicide rate in their countries by one-third by 2030 (2). However, no study at large scale to date has assessed the Suicidal Ideation among refugees in Ethiopia. Therefore, understanding those facts would give evidence for tackling the burden of Suicidal Ideation among refugees in Ethiopia and is essential to meeting WHO Mental Health Action Plan. The present study will assess the suicidal ideation among adult Eritrean Refugees in Tigray, Ethiopia.

## Methods

A community-based cross-sectional study was conducted among adult Eritrean refugees from February 2020 to April 2020. The study was conducted in the Mai-Aini Eritrean refugee camp, North West Tigray, Northern, which is located at a distance of 367 km from Mekele, the capital city of Tigray Regional State and 1,150 km from Addis Ababa, the capital city of Ethiopia. As of December 2018, the Eritrean refugee camp had a total population of 6,311 men and 5,826 women, of whom some 6,830 were adults aged 18–60 years (3,850 males, 2,980 females). There is one health center, jointly funded by the United Nations High Commissioner for Refugees (UNHCR) and the Agency for Refugees and Returnees Affairs (ARRA), providing several health services including mental health services.

The study participants included in the study were adult refugees aged between 18 and 60 years old who are living permanently in the Mai-Aini refugee camp. We excluded immigrants with impaired hearing and seriously ill refugees. The sample size was determined using a single proportion calculation formula assuming the following parameters: 50% proportion of participants with a suicidal ideation, 95% CI (Z1-α/2) = 1.96), 5% degree of marginal error (d), and 10% non-response rate. Given that the source population is below 10,000, we applied a correction formula, and the minimum required sample size was 400. A systematic random sampling technique was employed to recruit study respondents. We choose the next immediate house when there is no eligible participant in the selected household. When two or more eligible participants were present, we selected one eligible participant using the lottery method. The Geneva Convention defines a “refugee” as “someone unable or unwilling to return to their country of origin owing to a well-founded fear of being persecuted for reasons of race, religion, nationality, membership of a particular social group, or political opinion” (14).

The response variable in the study included suicidal ideation. Suicidal ideation was operationally defined using the World Mental Health (WMH) Survey Initiative Version of the World Health Organization (WHO) Composite International Diagnostic Interview (CIDI). The tool assessed the 12-month prevalence of suicidal behavior using the following question (14): “Have you thought of taking your life in the past 12 months?” with response options “yes (1)” i.e. I had any thoughts of suicide), and “No (2)” i.e., I haven't had any thoughts of suicide.

Beck Hopelessness Scale designed to measure three major aspects of hopelessness: feelings about the future, loss of motivation, and expectations. The test is designed for adults and consists of a list of 20 statements. The person is asked to decide about each sentence whether it describes his/her attitude for the last week including that day. If the statement is false for him, he should write false next to it. If the statement is true for him, he should write true next to it. Scores 4–8 indicate mild hopelessness, 9–14 moderate and 15–20 severe hopelessness. The translation of the BHS was accomplished according to the internationally accepted way of scientific measures (15). PTSD is a 4-item screen that was designed for use in primary care and other settings and is currently used to screen for PTSD includes an introductory sentence to cue respondents to traumatic events results of the PC-PTSD should be considered “positive” if a patient answers “yes” to any 3 item (16). Social support measure- was measure using three items Oslo Social Support scale as “poor,” “intermediate,” “good.” A sum index may also be made by summarizing the raw scores, the sum ranging from 3 to 14. A score of 3–8 is “poor support,” 9–11 is “moderate support” and 12–14 is “strong support” (17).

Data were collected using an interviewer-administered and structured questionnaire from the WHO survey questions. The questionnaire includes Socio-demographic and clinically related variables, psychosocial characteristics, and Substance use. To establish face validity and translation quality, the questionnaire was tested on 5% of the total sample size determined for this study, on participants outside the study site, by data collectors and supervisors during training. A few questions, language clarity, and information were revised and the questionnaire was finalized for the study. Three data collectors and supervisors were recruited and they were given rigorous training for 2 days. The supervisors followed the process of data collection, checked the consistency of data completeness, and communicated with the principal investigators on a daily basis.

Data were entered into Epi-info 7 and then exported to SPSS version 20.00 for analysis. We performed descriptive and inferential statistics. Tables, statements, charts, and graphs were used to present the findings of the analyzed data. Bivariate logistic regression analysis was conducted to see the crude association between the factors and outcome variables and select candidate variables (variables with *p*-value <0.2) to multivariable logistic regression. Adjusted odds ratio with 95% of CI was calculated and *P*-values <0.05 in the multivariable logistic regression were considered as a significant association.

## Results

### Socio-Demographic Characteristics

A total of 396 respondents have participated in the study making a response rate of 99%. The mean age of the respondent was 26.6 (+8.6) with minimum and maximum age were 18 and 60 years, respectively. Females contributed to the majority of 125 (55.1%) of the participants, and 215 (54.4%) participants were Christian Orthodox followers. Around 271 (68.4%) of respondents lived in rural areas before displaced from their country of origin. Also, the majority of the respondents, 206 (52%) attended primary education. The majority of the participant 312 (78.8%) were unemployed and 217 (54.8%) of the respondents stayed in the camp for <5 years. Table 1 demonstrates the socio-demographic characteristics of the study.

**Table 1:** Socio-demographic characteristics of respondents among adults age 18–60 years in Mai-Aini Eritrean refugee camp, Tigray, northern Ethiopia.

**Characteristics**		**Number**	**Percent**
Age	18–25	149	37.6%
	25–49	232	58.6%
	>50	15	3.8%
Sex	Male	204	51.5%
	Female	192	48.5%
Residential address	Urban	125	31.6%
	Rural	271	68.4%
Religion	Orthodox	215	54.4%
	Muslim	69	17.4%
	Protestant	58	14.6%
	Catholic	54	13.6%
Marital status	Married	198	50.0%
	Single	66	16.7%
	Divorced	132	33.3%
Educational status	No formal education	47	11.9%
	Primary	206	52.0%
	Secondary	95	24.0%
	Diploma and above	48	12.1%
Occupational status	Employed	84	21.2%
	Unemployed	312	78.8%
Length of stay	<5 years	217	54.8%
	>5 Years	179	45.2%
Ethnicity	Tigrigna	365	92.2%
	Saho	31	7.8%

### Clinical Variables

Table 2 describes the clinical variables. The majority of the respondents have no history of trauma [304 (76.8%)], have no history of a natural disaster [360 (90.9%)], and have no history of major family loss [290 (73.2%)]. On the other hand, 73 (18.4%) respondents had PTSD symptoms, 125 (31.6%) had depression symptoms, 45 (11.4%) respondents had a history of chronic illness, and 45 (11.4%) had a family history of mental disorders.

**Table 2:** Clinical related characteristics of respondents of adults age 18–60 years in Mai-Aini Eritrean refugee camps, Tigrai, northern Ethiopia.

**Characteristics**		**Number**	**Percent**
History of trauma	Yes	92	23.2%
	No	304	76.8%
History of natural disaster	Yes	36	9.1%
	No	360	90.9%
Major family loss or death of a loved one	Yes	106	26.8%
	No	290	73.2%
History of chronic illness	Yes	45	11.4%
	No	351	88.6%
Family history of mental illness	Yes	45	11.4%
	No	351	88.6%
PTSD symptoms	Have PTSD	73	18.4%
	No PTSD	323	81.6%
Depression symptoms	Depressed	125	31.6%
	Not depressed	271	68.4%

### Psychosocial and Substance Use-Related Factors

The majority of the respondents had no history of hopelessness [227 (57.3%)], had not lonely [281 (71%)], and had strong social support [234 (59.1%)]. Besides, 66 (16.7%) respondents had a history of ever use of alcohol drinking, and 47 (11.9%) respondents had ever use cigarette smoking in their lifetime in the camp (Table 3).

**Table 3:** Psychosocial and substance use-related characteristics of respondents in Mai-Aini, Eritrean refugee camps, Tigray, northern Ethiopia, 2020.

**Characteristics**		**Number**	**Percent**
Loneliness	Lonely	115	29%
	Not lonely	281	71%
Hopelessness	No hopelessness	227	57.3%
	Mild hopelessness	60	15.2%
	Moderate hopelessness	69	17.4%
	Sever hopelessness	40	10.1%
Social support	Poor social support	102	25.8%
	Moderate social support	60	15.2%
	Strong social support	234	59.1%
Worried about family back home	Yes	382	96.5%
	No	14	3.5%
Ever use of alcohol drinking	Yes	66	16.7%
	No	330	83.3%
Ever use of chat using	Yes	12	3.0%
	No	384	97.0%
Ever use of cigarette smoking	Yes	47	11.9%
	No	349	88.1%
Current use of cigarette smoking	Yes	33	8.3%
	No	363	91.7%
Current use of alcohol drinking	Yes	19	4.8%
	No	377	95.2%
Current use of chat chewing	Yes	5	1.3%
	No	391	98.7%

### Prevalence of Suicidal Ideation

Among the total of 396 adult refugees who participated in this study, 81 (20.5%) had suicidal ideation.

### Factors Associated With Suicidal Ideation

We found that history of trauma, history of chronic illness, family history of mental disorder, history of PTSD, were significantly associated with suicidal ideation (Table 4). Participants with a history of trauma had 2-fold [AOR = 2.3, 95% CI: 1.4, 4.5] higher odds of suicidal ideation than those who did not a history of trauma.

**Table 4:** Determinants of suicidal ideation, at Mai Aini, Eritrean refugee camps, Tigray, northern Ethiopia, 2020.

**Variables**	**Category**	**Suicidal ideation**		**COR (95% CI)**	**AOR (95%CI)**
		**Yes (%)**	**No (%)**		
Age in years	18–25	19 (23.5)	130 (41.3)	1.00	1.00
	26–49	57 (70.4)	175 (55.6)	2.3 (1.3, 3.9)^*^	1.7 (0.8, 3.8)
	>49	5 (6.2)	10 (3.2)	3.4 (1.1, 11.1)^*^	2.1 (0.5, 9.5)
Marital status	Married	37 (45.7)	161 (51.1)	1.00	1.00
	Divorced	19 (23.5)	47 (14.9)	1.7 (0.9, 3.3)^*^	1.2 (0.6, 2.8)
	Single	25 (30.9)	107 (34)	1.02 (0.6, 1.8)^*^	1.2 (0.6, 2.7)
History of trauma	No	47 (58)	257 (81.6)	1.00	1.00
	Yes	34 (42)	58 (18.4)	3.2 (1.9, 5.4)^*^	2.3(1.4, 4.5)^**^
History of natural disaster	No	67 (82.7)	293 (93)	1.00	1.00
	Yes	14 (17.3)	22 (34)	2.8 (1.4, 5.7)^*^	1.2 (0.4, 3.3)
Death of a lovely family	No	51 (63)	239 (75.9)	1.00	1.00
	yes	30 (37)	76 (24.1)	1.9 (1.1, 3.1)^*^	0.9 (0.5, 1.8)
History of chronic illness	No	58 (71.6)	293 (93)	1.00	1.00
	Yes	23 (28.4)	22 (34)	5.3 (2.8, 10.1)^*^	2.9 (1.3, 6.5)^**^
Family history of mental disorder	No	58 (71.6)	293 (93)	1.00	1.00
	Yes	23 (28.4)	22 (34)	5.3 (2.8, 10.1)^*^	3.08 (1.3, 7.06)^**^
History of PTSD	No PTSD	41 (50.6)	282 (89.5)	1.00	1.00
	Have PTSD	40 (49.4)	33 (10.5)	8.3 (4.7, 14.7)^*^	5.713 (2.8, 11.5)^**^
Level of depression	Not depressed	50 (61.7)	221 (70.2)	1.00	1.00
	Depressed	31 (38.3)	94 (29.8)	1.5 (0.9, 2.4)^*^	5.7 (2.8, 11.5)
Level of hopelessness	No hopelessness	49 (60.5)	178 (56.5)	1.00	1.00
	Mild hopelessness	6 (7.4)	54 (17.1)	0.4 (0.2, 0.9)^*^	0.4 (0.1, 1.4)
	Moderate hopelessness	11 (13.6)	58 (18.4)	0.7 (0.3, 1.4)^*^	0.9 (0.3, 2.01)
	Sever hopelessness	15 (18.5)	25 (7.9)	2.2 (1.07, 4.5)^*^	2.03 (0.9, 4.9)
Ever smoking cigarette	No	66 (81.5)	283 (89.8)	1.00	1.00
	Yes	15 (18.5)	32 (10.2)	2.01 (1.03, 3.9)^*^	2.7 (0.7, 10.4)
Current smoking cigarette in last month	No	70 (86.4)	293 (93)	1.00	1.00
	Yes	11 (13.6)	22 (34)	2.09 (0.9, 4.5)^*^	0.9 (0.2, 4.5)

N.B ^*^Significant by binary logistic regression at p-value < 0.2.

** *Significant by both binary and multiple logistic regressions at p-value < 0.05 and cut point at p-value < 0.05*.

The odds of having suicidal ideation were three times as much among those with a history of chronic illness than those without [AOR = 2.9, 95% CI: 1.3, 6.5]. Participants with a family history of mental disorder had three times [AOR = 3.1, 95% CI: 1.3, 7.1] more likely to have suicidal ideation than those with no family history of mental disorder. The odds of having a history of PTSD symptoms were five times [AOR = 5.7, 95% CI: 2.8, 11.5] more likely to have suicidal ideation than those who had no history of PTSD.

## Discussion

The present study determined the prevalence and risk factors for suicidal ideation among adult Eritrean refugee camps in Ethiopia. Eighty one (20.5%) of refugees reported suicidal ideation respectively. The prevalence of suicidal ideation in the present study was in line with a study conducted in North Korean refugees, Nigeria reporting 23.3% (18) but as different as compared findings from Germany (36%) (19), USA (5.3%) (4) and China (3%) (20).

Similarly, the prevalence of suicidal attempt was in line with a study conducted in Germany (7.6%) (19) but different when compared with findings from studies in Sri Lanka (11.9%) (21). These discrepancies could be argued due to the stability (or other negative experiences acquired from) of the country origin of the refugees, differences in expanded health service provision in the refugee camp, level of educational status of refugees, and degree of their spirituality or religiosity of refugees.

There are several predictive variables for suicidal ideation in our study. Our study revealed that participants who had a history of trauma were two times more likely to have suicidal ideation as compared to those who had no trauma, and this was inconsistent with finding from the USA (4, 22), Greek (23), and Europe (24). Such a history of trauma, which could be the resulted due to previous emotional abuse, physical abuse, sexual abuse, and neglect, may be mediated by depression, anxiety, and lack of perceived social support to cause suicidal ideation (25). It is important to design or strengthen interventions to manage the form of abuse mentioned above and mitigate the mental illnesses caused as a result of these forms of abuse. Concerning this, the current study found that participants who had a family history of mental disorder and history of PTSD were more likely to have suicidal ideation as compared to those who had not, as also found in Thailand, Sweden, Canada, and the US (26–29).

Consistent to a study from Jordan, USA, and Sweden (13, 30–32) the present study also found that participants who had a history of chronic illness were more likely to have suicidal ideation as compared to those who had no chronic illness. Karasouli et al. (33) explored how chronic illnesses could lead to suicidal ideation. They found that people with chronic physical illness suffer from pain, disfigurement, disability, and an invasive treatment regimen. This leads to having a compromised quality of life to the extent of unbearable pain or living with the illness intolerable, and these patients may deliberately not complain to their medication which could subsequently consider suicidality as a very real possibility (33). Health workers should be alert to intentional non-adherence as it may end one's life. It is crucial to recognize such signs and symptoms of hopelessness or demoralization and understand the suffering of people in clinical practices to develop suicide prevention programs.

The study has a few limitations. Given the quantitative nature of the study, data on sensitive issues or topics may be distorted due to some cultural grounds and self-reported nature of information on the study. Furthermore, the potential reasons and mediators leading to suicidal ideation may not be explored. The use of a cross-sectional approach also means that causal relationships cannot be determined. The respondents not assured that their answers would be treated with confidence and have felt that reporting their use of alcohol, chat, or smoking would harm their standing in any way.

## Conclusions

We found the following predictors for suicidal ideation: the history of trauma, history of chronic illness, family history of mental disorder, and history of PTSD was significantly associated with suicidal ideation. The following recommendations must be important to improve the rate of suicidal ideation: (i) provide ongoing professional development and training to healthcare workers to identify signs of mental disorders, PTSD, and demoralization/hopelessness to provide appropriate services or referrals; (ii) Integrating screening to all service provision site mental health disorders (PTSD, depression, chronic illness) in the outpatient departments (OPDs); (iii) a comprehensive multi-sectorial suicide prevention strategy is needed; and (iv) providing health information and awareness about the causes or factors of suicidal ideation in schools and other working offices on regular basis.

## Data Availability Statement

All data relevant to the study are included in the article or uploaded as online supplemental information.

## Ethics Statement

An Ethical Clearance was obtained from the Institute of Public Health, College of Medicine and Health Sciences, Aksum University College of Health Science (CHS). A permission letter was obtained from the zonal office and refugee camp before data collection. A letter of cooperation from kebeles administrators was also secured. Finally, written and verbal informed consent was obtained from every study participant included in the study during data collection time after explaining the objectives of the study and the right to withdraw from the study at any time. The patients/participants provided their written informed consent to participate in this study. Written informed consent was obtained from the individual(s) for the publication of any potentially identifiable images or data included in this article.

## Author Contributions

TG and MB conceived and designed the study. TG performed the statistical analysis and drafted the article. MB, TT, HG, and PW participated in article writing. All authors read and approved the final article.

## Conflict of Interest

The authors declare that the research was conducted in the absence of any commercial or financial relationships that could be construed as a potential conflict of interest.

## Publisher's Note

All claims expressed in this article are solely those of the authors and do not necessarily represent those of their affiliated organizations, or those of the publisher, the editors and the reviewers. Any product that may be evaluated in this article, or claim that may be made by its manufacturer, is not guaranteed or endorsed by the publisher.

## Abbreviations

ARRA: Agency for Refugees and Returnee Affairs
CVT: Center of Victim and Trauma
IDP: Internally Displaced Persons
IRC: International Rescue Committee
JRS: Jesuit Refugee Services
PTSD: Post-Traumatic Stress Disorder
USAID: United States Agency for International Development
UNFPA: United Nations Fund for Population Activities
UNHCR: United Nations Higher Commissioner for Refugee.
